# Combining biomedical knowledge graphs and text to improve predictions for drug-target interactions and drug-indications

**DOI:** 10.7717/peerj.13061

**Published:** 2022-04-04

**Authors:** Mona Alshahrani, Abdullah Almansour, Asma Alkhaldi, Maha A. Thafar, Mahmut Uludag, Magbubah Essack, Robert Hoehndorf

**Affiliations:** 1 National Center for Artificial Intelligence (NCAI), Saudi Data and Artificial Intelligence Authority (SDAIA), Riyadh, Saudi Arabia; 2 College of Computers and Information Technology, Taif University, Taif, Saudi Arabia; 3 Computer, Electrical and Mathematical Sciences and Engineering Division (CEMSE), Computational Bioscience Research Center (CBRC), King Abdullah University of Science and Technology (KAUST), King Abdullah University of Science and Technology, Thuwal, Saudi Arabia

**Keywords:** Biomedical literature, Biomedical knowledge graphs, Drug-target interactions, Drug-indications, Multi-modal learning, Bio-ontologies, Linked Data

## Abstract

Biomedical knowledge is represented in structured databases and published in biomedical literature, and different computational approaches have been developed to exploit each type of information in predictive models. However, the information in structured databases and literature is often complementary. We developed a machine learning method that combines information from literature and databases to predict drug targets and indications. To effectively utilize information in published literature, we integrate knowledge graphs and published literature using named entity recognition and normalization before applying a machine learning model that utilizes the combination of graph and literature. We then use supervised machine learning to show the effects of combining features from biomedical knowledge and published literature on the prediction of drug targets and drug indications. We demonstrate that our approach using datasets for drug-target interactions and drug indications is scalable to large graphs and can be used to improve the ranking of targets and indications by exploiting features from either structure or unstructured information alone.

## Introduction

Over the recent years, knowledge graphs have become an effective data model to store, retrieve, share and link domain-specific knowledge in healthcare and biomedicine (Bizer, Heath & Berners-Lee, 2011; Berners-Lee, Hendler & Lassila, 2001). Knowledge graphs refer to a form of knowledge representation that describes entities and the binary relations in which they stand (Paulheim, 2017; Ehrlinger & Wöß, 2016). Biomedical data from structured databases is often represented in the form of knowledge graphs, for example using the Resource Description Framework (RDF) (Brickley & Guha, 2004) as a way to link and cross-reference different databases (Jupp et al., 2014b; The UniProt Consortium, 2016). However, voluminous biological and biomedical scientific findings are recorded in the form of disparate unstructured knowledge available as free text in journals, papers, book chapters, *etc.*, with only a limited amount of curated information available in public databases. PubMed database alone stores more than 32 million research abstracts from biomedical and life sciences, while PubMed Central (PMC) provides free full-text access for about 7.3 million articles. Knowledge graphs embedding methods have emerged as a novel paradigm for analyzing and learning from knowledge graphs within and across different subject domains (Nelson et al., 2019; Ali et al., 2018; Alshahrani, Thafar & Essack, 2021). Several methods have been developed for information represented as graphs (Perozzi, Al-Rfou & Skiena, 2014), knowledge graphs (Ristoski & Paulheim, 2016; Nickel et al., 2016), or formal knowledge bases (Gutiérrez-Basulto & Schockaert, 2018). The key idea is to map knowledge graph entities and their relations into a vector representation which preserves some local structure of individual nodes, and possibly some global structure of the graph, and use the resulting representations in machine learning tasks such as link prediction, entity classification, relation extraction, and entity resolution (Nickel et al., 2016). Machine learning models developed using these methods can perform comparatively to traditional predictive methods that rely on manual feature engineering (Alshahrani et al., 2017).

Learning representations of entities is not restricted to entities retrieved from structured databases; representation learning has been applied to many other types of data such as text, images, or videos (LeCun, Bengio & Hinton, 2015). Word2Vec (Mikolov et al., 2013) or GLOVE (Pennington, Socher & Manning, 2014) can learn representations of words that preserve some word semantics under certain vector operations and can, therefore, be utilized for downstream analysis. Knowledge graphs are also used in the development of many natural language processing (NLP) systems (Xie et al., 2016; Hoffmann et al., 2011), where they provide background knowledge for purposes such as disambiguating word mentions (Dietz, Kotov & Meij, 2018). Computationally predicting new drug-target interactions (DTI) and drug indications is a challenge in drug repurposing that relies on information in several knowledge bases, such as Bio2RDF (Belleau et al., 2008), UniProt (Jupp et al., 2014a), and others (Williams et al., 2012). It has become more common to predict new uses for known drugs (*i.e.,* drug repurposing) using the information in such databases combined with information derived from in *silico* cheminformatics and structural bioinformatics methods (Chen et al., 2015; Pryor & Cabreiro, 2015). A recent example of computational drug repurposing for COVID-19 used graph techniques to identify six drugs (Gysi et al., 2021). All six drugs exhibit the ability to reduce viral infections experimentally. Moreover, four of the drugs show very strong anti–SARS-CoV-2 response, which suggests they can be repurposed to treat COVID-19 (Gysi et al., 2021). Overall, the computational approaches developed to predict DTI and drug indications (Ezzat et al., 2018; Thafar et al., 2019; Muñoz, Nováček & Vandenbussche, 2017; Mohamed, Nováček & Nounu, 2019) differ in the algorithms they employed and the data sources utilized. That is, the network-based approaches (*i.e.,* graph-based methods) developed for drug repurposing utilize different data sources, including genomic and chemical similarities and various other drugs and target interactions profiles or descriptors (Yamanishi et al., 2008; Wang et al., 2014a), integrate information related to drug mechanisms, and use machine learning techniques or graph inference methods to predict novel DTIs (Seal, Ahn & Wild, 2015; Thafar et al., 2020b; Fu et al., 2016; Chen et al., 2012; Thafar et al., 2020a; Thafar et al., 2021).

Graph embeddings applied on the knowledge graphs improves the DTI prediction performance through the learning of low-dimensional feature representation of drugs or targets, used with the machine learning models. For example, the recently developed DTINet (Luo et al., 2017) used graph embedding approaches and matrix factorization, to predict novel DTIs from a heterogeneous graph. DTINet combines different types of drug and target (*i.e.,* protein) information such as drug–disease associations, drug–side effect associations, drug–drug similarity, drug–drug interactions, protein–protein interaction, protein–disease associations, and protein–protein similarities to construct a full heterogeneous graph. Another recent example of a knowledge graph-based method, TriModel (Mohamed, Nováček & Nounu, 2019), formulates DTI prediction as a link prediction problem associated within a knowledge graph. It learns feature representations (*i.e.,* knowledge graph embeddings) for entities and relations from a knowledge graph that integrated information from multiple structured databases similar to DTINet, and then predicts novel DTIs based on their interaction scores calculated using trained tensor factorization applied on the knowledge graph embeddings.

Some other approaches to drug repurposing rely on integrating entities text-mined from the biomedical literature (unstructured text) into knowledge graphs to predict novel associations between drugs and targets or drugs and diseases (Swanson, 1990; Andronis et al., 2011; Frijters et al., 2010; Agarwal & Searls, 2008). One such example is the biomedical knowledge graph-based method, SemaTyP (Semantic Type Path) (Sang et al., 2018). SemaTyP predicts candidate drugs for diseases by text-mining entities in published biomedical literature. This method first constructed a semantic biomedical knowledge graph, SemKG, with extracted relations from PubMed abstracts, then a logistic regression model is trained by learning the semantic types of paths of known drug therapies existing in the biomedical knowledge graph. Finally, the learned model, SemaTyP, is applied to exploit the semantic types of paths to discover drug therapies for new diseases. SemaTyP is the first method focused on drug repurposing that uses entities text-mined from biomedical literature and knowledge graph to predict candidate drugs. Another such recent method focused on drug repurposing, GNBR (Global Network of Biomedical Relationships) (Percha & Altman, 2018), also uses a large, heterogeneous knowledge graph to leverage integrated biomedical information across the literature of pharmacology, genetics, and pathology. The GNBR knowledge graph is generated based on three types of entities (drugs, diseases, and target proteins) that are connected by semantic relationship derived from the biomedical literature abstracts. The embedding method applied to this knowledge graph explicitly models the uncertainty associated with literature-derived relationships. Thus, GNBR is the first method that incorporates uncertainty (*i.e.,* noise) into a literature-based graph embedding method, allowing for a more precise and nuanced drug repurposing model. The GNBR method for drug repurposing produced treatment hypotheses with strong evidence from published literature, evaluated using gold-standard drug indications. Furthermore, they applied their model to generate novel drug repurposing hypotheses and assess their scientific validity using a variety of sources.

Despite several methods extracting biological relations from text, data integration issues remain between knowledge graphs and biomedical literature. First, biological entities are mentioned in knowledge graphs and biomedical literature using different vocabularies and thesaurus, which leads to low coverage when integrating structured knowledge graphs and unstructured biomedical literature. We address this issue by utilizing bio-ontologies for normalizing and unifying mentions of biological entities at the token level. Another problem is that knowledge graph learning or text-based methods, when used alone, fail in the “zero-shot scenario” when an entity is absent from either the knowledge graph or the text corpus and therefore can not be seen during training. Also, both modes of representation lack automatic feature generation. This work presents a method that combines knowledge graphs with rich textual content in the scientific literature in a unified representation learning framework. Additionally, our approach addresses the issues mentioned above of low coverage and different mentions of biological entities by utilizing bio-ontologies for normalization at the token level. We also tackle the ”zero-shot scenario” through joint representation learning between knowledge graphs and literature. The primary goal is to complement the knowledge graph representation model presented previously with the model that utilizes background knowledge of biological entities available in the biomedical literature. We demonstrate that this multimodal view of feature representation enhances the prediction results of biological relations such as drugs targets and indications.

## Materials and Method

### Data sources and benchmark datasets

**Figure 1:**
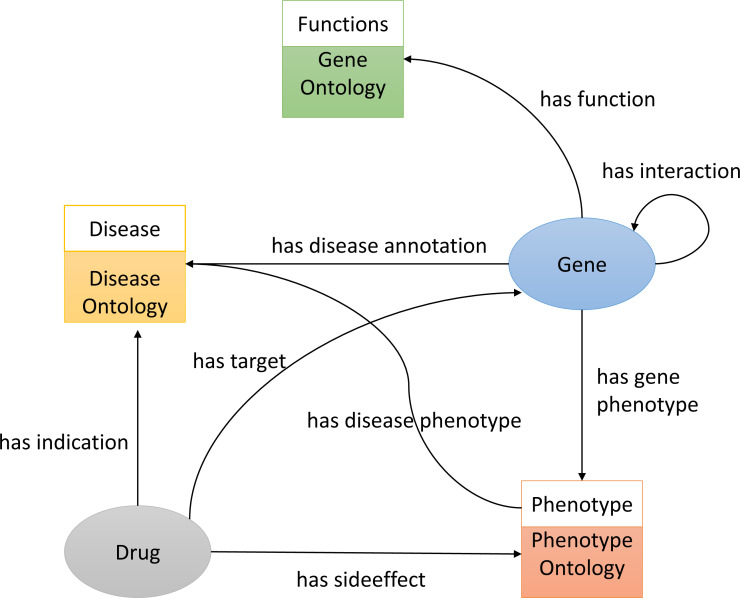
An illustration of the knowledge graph used to repurpose the drugs. To predict drug targets, we removed all the *has-target* links in the graph before applying our random walk algorithm. Similarly, for predicting drug indications, we removed all the *has-indication* links in the graph before applying our random walk algorithm.

To construct the knowledge graph we used three ontologies, Gene Ontology(GO) (Ashburner et al., 2000), Disease Ontology (DO) (Schriml et al., 2011), and the Human Phenotype Ontology (HPO) (Köhler et al., 2014) (see Fig. 1). It also includes several biological entities such as diseases, genes (we do not distinguish between genes and proteins in the graph), and chemicals/drugs. The graph further includes relations between entities such as the protein-protein interactions obtained from STRING (Szklarczyk et al., 2010) (file: protein.actions.v10.txt.gz), chemical–protein interactions from STITCH (Kuhn et al., 2012) (file: 9606.actions.v4.0.tsv), and drugs and their side-effects and indications from SIDER (Kuhn et al., 2015) (file: meddra_all_indications.tsv). We downloaded all the above-mentioned data on 11 March 2018 and used it to build the knowledge graph using RDF.

For the text-derived corpus 2, we used the pre-annotated Medline corpus provided by the PubTator project (Wei, Kao & Lu, 2013), downloaded on 18 December 2017. PubTator is a web-based tool designed to assist manual biocuration (*e.g.*, annotating biological entities and their relationships) through the use of advanced text-mining techniques. This corpus contains 27,599,238 abstracts together with annotations for chemicals, genes/proteins, and diseases. PubTator has annotations for 17,505,118 chemicals that represent 129,085 distinct drugs using either CHEBI or MESH identifiers. PubTator also contains 17,260,141 gene mentions covering 137,353 distinct genes in different species, of which 35,466 refers to human genes. We used 9,545 of the STITCH identifier (using the file 9606.protein.aliases.v10.txt provided by STITCH). PubTator further contains 81,655,248 diseases that represent 8,143 distinct diseases in MESH. We used DO to map diseases to 2,581 distinct DO classes. Table 1 provides the statistics for the DTI and drug indication data used to evaluate the models.

Additionally, we added gold standard datasets Table 2 commonly used in the literature to evaluate DTI prediction methods, *i.e.,* the Yamanishi (Yamanishi et al., 2008) and DrugBank datasets (Wishart et al., 2008). The Yamanishi dataset consists of interactions of drugs with four types of proteins, namely: Enzyme (E), Ion Channel (IC), G-protein-coupled receptor (GPCR), and Nuclear receptor (NR). We utilized the Enzyme and Ion Channel groups as they contain the largest number of interactions and can be found in our graphs after mapping of drugs ID (KEGG IDs) to our graph IDs (PubChem IDs).

### Knowledge graph construction

We build the RDF graph by linking biological entities with relations from each database. For example, we link drug and protein targets from STITCH by the *has target* relations. The relations between the different biological entities are shown in Fig. 1. We also added classes from GO, HPO and DO onologies. For example, we link the disease *primary pulmonary hypertension* (DOID:14557) to the phenotype *arrhythmia* (HP:0011675) (using a has phenotype relation), we link the gene *CAV1* to disease * primary pulmonary hypertension* (DOID:14557) (using a has disease association relation), and we link the drug *Tadalafil* (CID00110635) to phenotype *abdominal pain* (HP:0002027) (using a has side effect relation), as well as disease *connective tissue disease* (DOID:65) (using a has indication relation):


 
 
@prefix doid: <http://purl.obolibrary.org/obo/DOID_> . 
@prefix hp:  <http://purl.obolibrary.org/obo/HP_> . 
@prefix b2v: <http://bio2vec.net/relation/> . 
@prefix entrez: <http://www.ncbi.nlm.nih.gov/gene/> . 
@prefix stitch: <http://bio2vec.net/CID> . 
doid:14557 b2v:has_disease_phenotype hp:0011675 . 
entrez:857 b2v:has_disease_association doid:14557. 
stitch:00110635 b2v:has_sideeffect hp:0002027 . 
stitch:00110635 b2v:has_indication doid:65 .    


### Integrating structured biomedical knowledge and literature

We use RDF (Beckett, 2004) to express and integrate structured information considered to be useful for predicting DTI and drug indication associations. In RDF, knowledge is expressed in a graph-based format in which entities (*i.e.,* nodes) are represented by an Internationalized Resource Identifier (IRI), and the relations between entities are represented as edges (*i.e.,* an edge connects two entities). Specifically, to integrate several datasets related to drug actions and diseases in a knowledge graph using RDF as representation language, we combine information about drugs and their targets (Kuhn et al., 2012) and indications (Kuhn et al., 2015), gene–disease associations (Piñero et al., 2016), and disease phenotypes (Hoehndorf, Schofield & Gkoutos, 2015), as well as gene functions and interactions between gene products (Szklarczyk et al., 2010). We further added biological background knowledge expressed in the HPO, GO, and DO ontologies, directly to this RDF graph so that the superclasses of phenotypes can be accessed and used by the machine learning model.

**Table 1:** Statistics of the datasets used in model training and evaluation.

	**Overlap in KG and literature**		**Training data (80%)**		**Testing data (%)**	
**Dataset**	**No. of drugs**	**No. of targets or (of diseases)**	**No. of positive samples**	**No. of negative samples**	**No. of positive samples**	**No. of negative samples**
Drug–target interactions	820	17,380	65,379	65,379	16,345	16,345
Drug–indication associations	754	2,552	6,363	6,363	1,591	1,591

**Table 2:** Statistics of the datasets.

**Dataset**	**No. drugs**	**No. of targets**	**No. of positive assoc.**	**No. of unknown assoc.**
**Enzyme (E)**	445	664	2926	292,554
**Ion Channel (IC)**	210	204	1476	41,364
**Drugbank dataset**	1,482	1,408	9881	2,076,775

**Figure 2:**
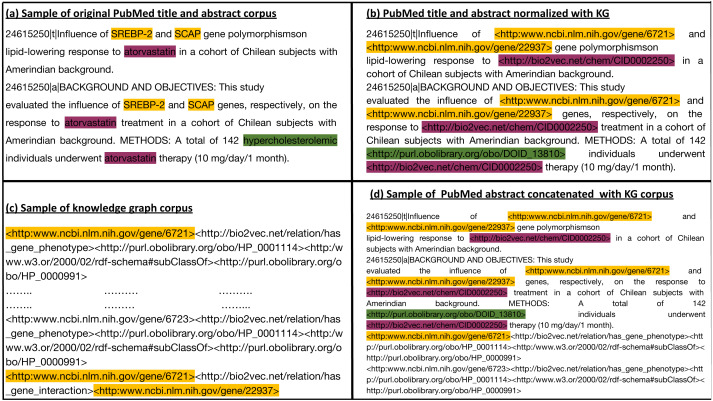
(A) Sample of the original Pubmed title and abstract; (B) Illustration of how we normalize literature abstracts to our knowledge graph to ensure that both overlap on the level of tokens. It shows the use of ontologies to normalize synonymous or similar terms to their respective ontology identifiers as in *hypercholesterolemic*. We refer to NCBI semantic web links for genes. For other entities with no standard semantic web links, we assign them to links that start with http://bio2vec.net/. (C) Sample of the knowledge graph corpus. (D) Sample of the knowledge graph corpus concatenated with the PubMed abstract corpus.

We generate a corpus from the RDF graph by applying iterated random walks (Alshahrani et al., 2017). We considered each random walk as a sequence that expresses a chain of statements following a random path through the knowledge graph. Subsequently, we align the entities that occur in our knowledge graph with the information contained in the biomedical literature. For this purpose, we normalized the entities in the abstracts of the biomedical literature to the entities in the knowledge graph using named entity recognition and entity normalization approaches (Rebholz-Schuhmann, Oellrich & Hoehndorf, 2012). Specifically, we normalized the drug, gene, and disease names/symbols to the knowledge graph using the annotated literature in PubMed abstracts provided by the PubTator (Wei, Kao & Lu, 2013) database, and the mappings provided between different vocabularies of drugs and diseases. PubTator aggregates different entity normalization approaches such as GNorm (Wei, Kao & Lu, 2015) or DNorm (Leaman, Islamaj Doğan & Lu, 2013), which can also be used directly with new text. We then processed the annotated PubMed abstract corpus by replacing each entity (*i.e.,* gene, drug/chemical compound, or disease) with the IRI used to represent the synonymous entities in the knowledge graph. This replacement ensures that our literature entities and knowledge graph entities overlap on the token level. Figure 2 provides an illustration of the normalization step between literature entities and knowledge graph entities overlap. We then used the knowledge graph to generate corpus 1 using an edge-labeled iterated random walk of fixed length without restart (Alshahrani et al., 2017). For each node in the graph, we generated a sequence based on a short random walk, where each walk is a sequence of nodes and edges (refer to Table S1 for more information). We used two hyperparameters to generate the corpus: walk-length (the size of each walk sequence) and the number of walks (the total number of walks generated for each node).

**Figure 3:**
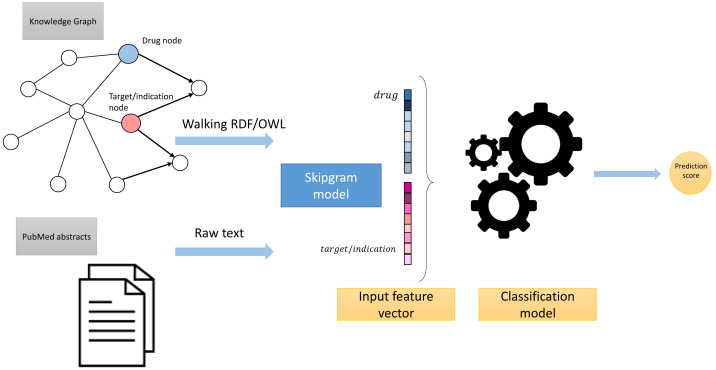
High-level overview of the workflow.

These processing steps led to the generation of two corpora: Corpus 1 generated from random walks starting from nodes in our knowledge graph, and Corpus 2 generated from annotated literature abstracts in which entities in the literature that also appear in our graph have been replaced by the IRI of the entities in the knowledge graph. These two corpora form the foundation of our feature learning step. Figure 3 provides an overview of the workflow.

### Generating embeddings

Word2Vec is a vector space model that maps words to vectors based on the co-occurrence of words within a context window across the text corpus. Thus, in our graph, these semantics are captured by the random walks representing the co-occurrence of different entities and relations. We used the Word2Vec skip-gram model (Mikolov et al., 2013) to generate embeddings for the corpus generated by random walks on the knowledge graph (corpus 1) and for the Medline corpus (corpus 2). For both corpora, we used negative sampling with 5 words drawn from the noise distribution, a window size of 10, and an embedding dimension of 128, based on the parameter optimization results. Additionally, we generate embeddings by using the TransE (Bordes et al., 2013) knowledge graph embedding method. TransE is an embedding model specifically designed for knowledge graphs; it leverages the translation in the vector space. That is, given Given a triple *(subject, predicate, object)* or simply *(s,p,o)*, it aims to make the sum of the subject and predicate vectors as close as possible to the object vector (*i.e.,* $\vec{s}+\vec{p}\approx \vec{o}$) when *(s,p,o)* holds, and the sum is far away otherwise. This is done based on some distance measure $d(\vec{s}+\vec{p},\vec{o})$, which is chosen to be *L*_1_ or *L*_2_ norms. The loss function is the pairwise ranking loss as follows:

(1) \begin{eqnarray*}\mathcal{L}=\sum _{(s,p,o)\in \mathcal{S}}\sum _{({s}^{{^{\prime}}},p,{o}^{{^{\prime}}})\in {\mathcal{S}}^{{^{\prime}}}}[\gamma +d(\vec{s}+\vec{p},\vec{o})-d({\vec{s}}^{{^{\prime}}}+\vec{p},\vec{{o}^{{^{\prime}}}})]\end{eqnarray*}

The TransE model deals with only one-to-one relations, but it fails to account for other types of relations and mapping properties such as one-to-many, many-to-one, and many-to-many which are mitigated by other knowledge graphs embeddings variants such as TransH (Wang et al., 2014b), TransR (Lin et al., 2015) and others (Ji et al., 2021).

### Training the prediction models

We evaluated the performance of each method by using the embedding vectors to predict DTI and drug indication associations in a supervised manner. For prediction models, we used neural networks-based models such as: Artificial neural networks (ANN) and Siamese Networks (Bertinetto et al., 2016). The Siamese network uses a unique structure to learn similarity between inputs even with the presence of one or training example and able to generalize to data from complete different distributions with new classes. Although they have been widely used for images, they could be also applied to learning similarity between any two different entities encoded as feature vectors. Moreover, we have used Random forests (RF), and logistic regression (LR) classifiers as basic and self-explained machine learning models. For each model, the dataset was randomly split into 80% and 20%. proportions for the training set and testing set, respectively. The models were trained as binary classification models to predict whether there is an interaction between drug and target or not (based on the drug-target dataset), or if there is an association between drug and disease or not (based on the drug indications dataset). Table 1 provides all the statistics for the DTI and drug indication data used to evaluate the models.

For ANN model training, we implemented an architecture with a single hidden layer that is twice the size of the input vector. We used the Rectified Linear Unit(ReLU) (Nair & Hinton, 2010) as an activation function for the hidden layer and a sigmoid function as the activation function for the output layer. We also used cross-entropy as the loss function, RMSprop optimizer (Hinton, Srivastava & Swersky, 2012) to optimize the ANN parameters, and we implemented all these steps using Keras library in Python (Gulli & Pal, 2017). We optimized the ANN architecture and the size of the embeddings using a narrow search (see Tables S2 and S3), we have also optimized the learning rate and the number of dense layers of the Siamese networks. To train the RF classifier, we specified the number of trees to be 50, with the minimum number of one for the training samples in leaf nodes, and used the Gini impurity index to measure the quality of the split. For the LR, we optimized the LR concerning two of its most effective hyperparameters: the penalty term [L1,L2] and the C = [100,10,1.0,0.1,0.01] (the inverse of regularization), which controls the strength of the penalty. Small values of this hyperparameter cause stronger regularization. We found that L2 and *C* = 10 are the optimal values. We trained the LR classifier using scikit-learn (version 0.17.1) in Python (Pedregosa et al., 2011).

## Results

### Learning and combining features

**Figure 4:**
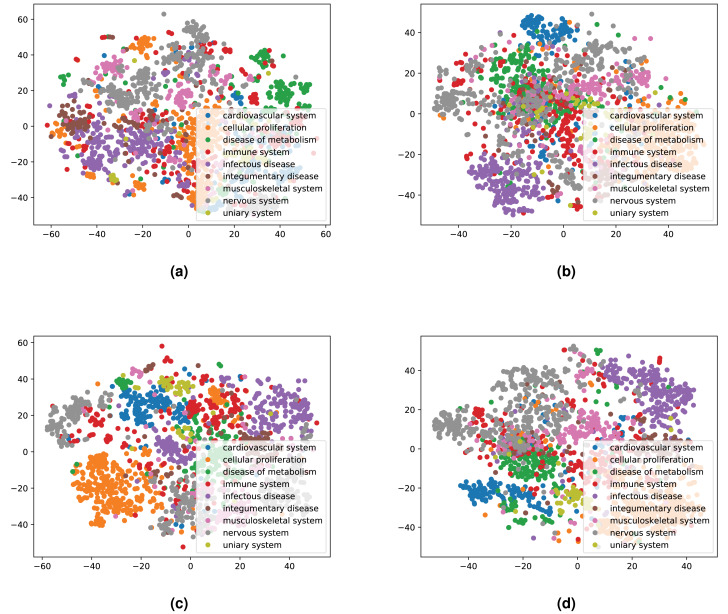
Illustrations of the 2D t-SNE plots for diseases based on different embeddings. (A) Knowledge graph. (B) MEDLINE abstracts. (C) Concatenated embeddings. (D) Concatenated corpora through jointly learned embeddings from literature and knowledge graph. The diseases are colored according to their top-level categories in the Disease Ontology.

**Figure 5:**
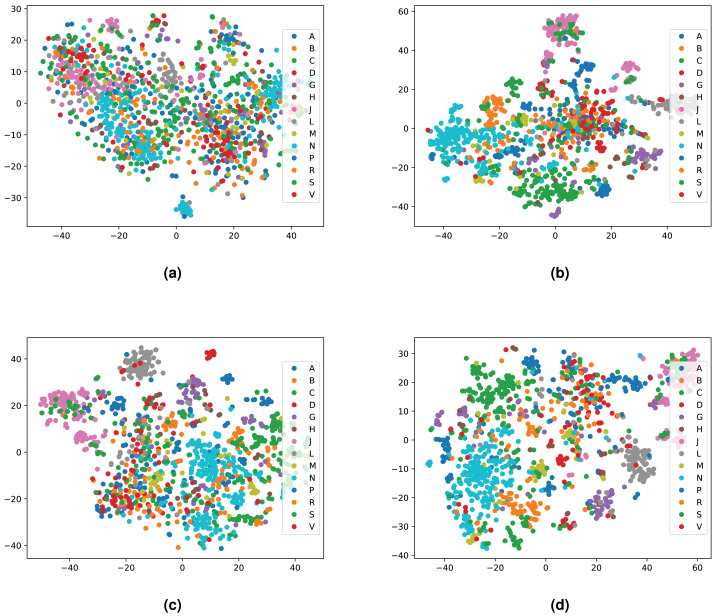
Illustrations of the 2D t-SNE plots for drugs based on different embeddings approaches. (A) Knowledge graph. (B) MEDLINE abstracts. (C) Concatenated embeddings. (D) Concatenated corpora through jointly learned embeddings from literature and knowledge graph. The drugs are colored according to their top-level ATC class.

We integrated both data sources intending to leverage the information in a single predictive model. To achieve this goal, we obtained embeddings for all entities. We used two embedding approaches for the knowledge graph including the Word2Vec skip-gram model (Mikolov et al., 2013), and TransE (Bordes et al., 2013), and for biomedical literature only the Word2Vec skip-gram model. We used two different approaches to combine the embeddings from corpus 1 and 2. First, we generated the embeddings for each corpus, then concatenated the embedding vectors from both corpus. Second, we concatenated the two corpora, then generated jointly-learned embeddings from the combined corpus. Here, it should be noted that not all entities in the knowledge graph have a representation in literature, and not all entities (drugs, diseases, and genes) mentioned in literature are included in the knowledge graph. Nonetheless, we obtained embeddings for all entities in corpus 2, in particular, for the entities which we normalized to our knowledge graph. Figure S1 shows the overlap between the two datasets. Figures 4 and 5 show a visualization of the embeddings (from the knowledge graph, literature, and combined) using t-SNE (Van der Maaten & Hinton, 2008). Disease embeddings are coloured based on their top-level DO class, and drug embeddings based on their top-level class in the Anatomical Therapeutic Chemical (ATC) Classification System. The clustering by both top-level DO classes and the top-level ATC categories shown in both figures indicate that the embeddings cluster into biologically meaningful groups.

### Evaluating the prediction performance

We evaluated the performance of our method by predicting drug-target interactions and drug indications. For this purpose, we used five different evaluation methods: (1) embeddings generated from the knowledge graph *via* the TransE model, (2) embeddings generated *via* the Word2Vec skip-gram model from the knowledge graph alone after the corpus generation through random walks (Walking RDF/OWL), (3) embeddings generated from the literature corpus alone *via* the Word2Vec skip-gram model, (4) concatenated embeddings from (2) and (3), and (5) jointly-learned embeddings generated from combining corpus 1 and corpus 2 on which we applied the Word2Vec skip-gram model. We used drug-target interactions from the STITCH database and drug indications from the SIDER database as evaluation datasets. Furthermore, to clearly distinguish and evaluate the contributions of the different data sources (*via* the five evaluation methods), we only used the entities in the evaluation dataset that have a representation in both the knowledge graph and the literature corpus. Before training the model, we removed all has-target edges (when predicting the drug-target interactions) or has-indication edges (when predicting drug indications) in the graph before generating the predictions for drug-target interactions and drug indications, respectively.

Consequently, from the drug-target interactions dataset, we obtained 81,724 positive samples, and from the drug indications dataset, 7,954 positive samples. For the negative samples, we randomly selected the same number of negative samples as positive samples from a massive number of negative samples that exist in the datasets. In this manner, we ensured a balanced dataset was used to develop the prediction models.

For predicting drug-target interaction, we used an evaluation set of 820 drugs that was mapped to 17,380 targets. For predicting drug indications, we used 754 drugs with one or more known indications and rank 2,552 diseases for each of the drugs to determine which disease it may treat (see Tables S4 and S5 for details about the counts in all resources). For each model, the input feature vector is the drug embedding concatenated with the target embedding, for the drug-target pairs. Similarly, for drug indications the input vector is the drug embedding concatenated with the disease embedding. The output indicates whether the drug interacts with the targets or the drug treats the disease. We evaluated the performance of each model, using 20% of associations left out of the training process. All three of our classification models can provide confidence values for a prediction, and we ranked predicted associations based on their confidence value. We then calculated the area under the receiver operating characteristic (ROC) curve (AUROC) (Fawcett, 2006), as well as the recall of each drug averaged among all drugs.

Furthermore, we have compared the results of using the four variants of our models (the knowledge graph, PubMed abstracts, Concatenated embeddings, and Concatenated corpus) with other benchmark datasets. Tables 3 and 4 shows the results in terms of AUROC and AUPR. We compared the results of our approach with five state-of-the-art methods, namely: BLM-NII (Mei et al., 2013), KRONRLS-MKL (Nascimento, Prudêncio & Costa, 2016), DNILMF (Li, Li & Bian, 2019), and the latest two methods: TriModel (Mohamed, Nováček & Nounu, 2020) and DTiGEMS+ (Thafar et al., 2020a). We observe that our models’ performance, which utilizes the multimodal approaches (Concatenated embeddings and Concatenated corpus), is not as competitive as the latest methods but shows comparable results (especially in Yamanishi datasets with the previous methods including BLM-NII, KRONRLS-MKL, and DNILMF), coming as the best and the second best performing models for the Ion Channel, while it comes as the second best in Enzyme dataset in the AUROC results analysis shown in Table 3.

**Table 3:** AUROC results of comparisons with other methods on Yamanishi (Enzyme and Ion Channel) and Drugbank datasets. Bold indicates best performing model while underline indicates second best performing model.

		Datasets	
Model	Enzyme	Ion channel	Drugbank
Ours (KG)	0.900	0.970	0.840
Ours (PubMed abstracts)	0.950	0.970	0.880
Ours (Concatenated embeddings)	0.940	** 0.990**	0.880
Ours (Concatenated corpus)	0.960	** 0.990**	0.890
BioBERT embeddings	0.920	0.880	0.900
BLM-NII	0.950	0.900	0.940
DNILMF	0.950	0.930	0.940
KRONRLS-MKL	0.920	0.890	0.920
TriModel	** 0.990**	** 0.990**	** 0.990**
DTiGEMS+	** 0.990**	** 0.990**	0.970

**Table 4:** AUPR results of comparisons with other methods on Yamanishi (Enzyme and Ion Channel) and Drugbank datasets. Bold indicates best performing model while underline indicates second best performing model.

		Datasets	
Model	Enzyme	Ion channel	Drugbank
Ours (KG)	0.690	0.920	0.280
Ours (PubMed abstracts)	0.740	0.900	0.320
Ours (Concatenated embeddings)	0.740	0.950	0.340
Ours (Concatenated corpus)	0.760	0.950	0.320
BioBERT embeddings	0.908	0.870	**0.879**
BLM-NII	0.830	0.800	0.110
DNILMF	0.820	0.840	0.410
KRONRLS-MKL	0.800	0.820	0.340
TriModel	0.930	0.950	0.670
DTiGEMS+	**0.960**	**0.960**	0.610

Additionally, we have employed BioBERT (Lee et al., 2020) embeddings, which is a domain-specific language model based on the BERT model (Devlin et al., 2018), pre-trained on large-scale biomedical text (PubMed abstracts and PMC full-text articles). For each drug and gene name, we have extracted their BioBERT embeddings. We used each pair of interacting drug-gene BioBERT embeddings as inputs to the Siamese network. We have followed the same approach of training and testing as described in Training the prediction models Tables 3 and 4 show the ROCAUC and AUPR scores on the three datasets we used for benchmarking namely (Drugbank, Yamanishi Enzyme and Yaminishi Ion channel). Tables S7 and S8 (Supplementary) summarizes our results for the prediction of DTIs and drug indications using different machine learning models.

While other methods may be limited to certain sizes of the graph, the main advantages of our models canbe summarized in the following points. first, Its scalability to large and massive knowledge graphs, our graph used in this work is ≈500 times larger than the Enzyme dataset, and ≈148 times larger than the Drugbank dataset. Second, Our models are generic and automatically learn the features, while other methods may rely on laborious feature extraction and manually engineered feature vectors. For example, DtiGems+ construct different types of graphs and compute many similarity scores such as drug–drug similarity, target–target similarity as well as adapting several techniques such as graph embeddings, graph mining as well as the use of machine learning models as downstream classifiers. Although these approaches resulted in improved prediction accuracy due the collective power of different types of features, they require domain-specific knowledge of manually-engineered features, incorporate complex processes of extraction and include many steps of data integration and graph infusions. This doesn’t fully utilize feature learning as an optimal, efficient, and elegant way of finding the most relevant features. lastly, our proposed models attempt to resolve the issues related to the low coverage in the knowledge graph and the textual content by utilizing bio-ontologies for entity normalization.

We found that both ANN and RF classifiers were able to accurately predict both DTIs and drug indications, while the LR classifier results in relatively worse performance. An obvious explanation is that LR mainly assigns weights to individual features and cannot compare or match elements of the two input embedding vectors, while both the ANN and RF classifiers can provide a classification based on comparing elements of the two input embedding vectors. Furthermore, we found that, in general, using embeddings generated from literature results in higher predictive performance across all classifiers compared to embeddings generated from the knowledge graph alone. Also, combining the embeddings, or using jointly learned embeddings sometimes but not always improves or changes the predictive performance.

Additionally, we examine the actual performance in terms of the ranking given by each of our embeddings approaches for a sample of drug–targets and drug–indications pairs. Table S7 (refer to Supplementary) shows the predicted rank number given for each approach for the prediction of drug–targets, while Table S10 (refer to Supplementary) shows the predicted rank number in drug–indications. We find that the combined approaches (Concatenated embeddings and Concatenated corpus) improved the predicted ranks over the performance of the knowledge graph and the PubMed abstracts alone.

**Figure 6:**
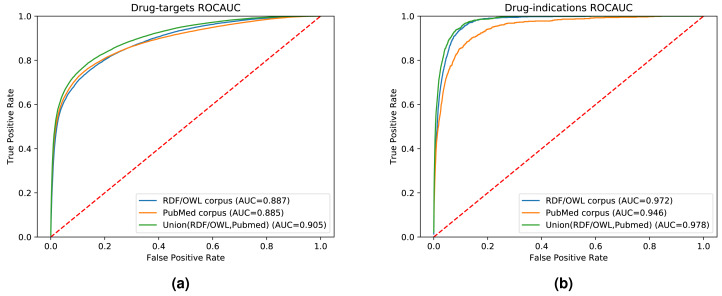
ROC curve of our neural network for predicting drug targets in the union of associations present in the knowledge graph and PubMed abstracts (left); ROC curve of our neural network for predicting drug indications found in the union of knowledge graph and PubMed abstracts (right).

While our results indicate that both literature-derived and knowledge graph embeddings can be used to predict interactions, the main contribution of our multi-modal approach is the increased coverage through combining database content and literature (see Fig. S1). We used the common drug, target, and disease entities between the knowledge graph and literature in the previous experimental setups. Here, we further quantify fairly the impact of the information provided by each data modality on the prediction performance. We also demonstrate the broader application of our method by extending our evaluation set to contain all the drugs, genes, and diseases found in either our knowledge graph, literature abstracts, or the union of the entities in the knowledge graph and literature trained on the combined corpus. Figure 6 shows the ROC curves and the AUROC for predicting DTIs and drug indications using ANN, based on a combination of the literature corpus and the random walk corpus.

Our knowledge graph contains a massive number of chemicals, many of which are not drug-like, and while the performance in predicting drug targets is somewhat higher when using the knowledge graph embeddings, the overall performance is still dominated by the literature-derived embedding vectors. However, when predicting indications for known drugs, both our graph and literature overlap more substantially while nevertheless containing complementary information. We observe a significant improvement in predicting drug indications when combining the information from literature and the knowledge graph. All DTI predictions, as well as the predictions for drug indications, are available at https://github.com/bio-ontology-research-group/multi-drug-embedding.

## Discussion

There are many scenarios in biological and biomedical research in which predictive models need to be built that can utilize information that is represented in different formats. Our key contribution is a method to integrate data represented in structured databases, in particular knowledge graphs represented in RDF and OWL, and integrate this information with information in literature. While we primarily focus on the prediction of DTIs and drug indications based on information in text and databases, our approach is generic and can serve as a paradigm for learning from multi-modal, heterogeneous data in biology and biomedicine.

Our method uses feature learning to project different types of data into a vector space, and combine data of different modes either within a single vector space (when mapping data of different modes to the same space, or to vector spaces of identical dimensions) or we combine the vector spaces themselves. We rely on the recent success of deep learning methods (Raví et al., 2017; Angermueller et al., 2016) which improved our ability to learn relevant features from a data set and project them into a vector space. In particular, our approach relies on natural language models, in particular Word2Vec (Mikolov et al., 2013), and recent approaches to project information in knowledge graphs into vector spaces (Nickel et al., 2016; Alshahrani et al., 2017). Furthermore, the use of supervised learning on feature vectors has been shown to improve classification performance over traditional techniques as they become accessible to build task-specific machine learning models (Smaili, Gao & Hoehndorf, 2018; Smaili, Gao & Hoehndorf, 2019). In this work, the classifiers we used utilize the similarity-based embeddings to learn decision boundaries between the two classes(*i.e.,* interacting/non-interacting relations). These approaches are now increasingly applied in biological and biomedical research (Alshahrani & Hoehndorf, 2018) yet often restricted to single types of representation (such as images, genomic sequences, text, or knowledge graphs).

Our approach naturally builds on the significant efforts that have been invested in the development of named entity recognition and normalization methods for many different biological entities (Rebholz-Schuhmann, Oellrich & Hoehndorf, 2012) as well as the effort to formally represent and integrate biological data using Semantic Web technologies (Jupp et al., 2014a; Callahan et al., 2013). Several biological data providers now provide their data natively using RDF (Jupp, Stevens & Hoehndorf, 2012; UniProt Consortium, 2018). Furthermore, many methods and tools have been developed to normalize mentions of biological entities in text to biological databases, for example for mentions of genes and proteins, (Leaman & Gonzalez, 2008; Wei, Kao & Lu, 2015), chemicals (Leaman, Wei & Lu, 2015) as well as diseases (Leaman, Islamaj Doğan & Lu, 2013), and repositories have been developed to aggregate and integrate the annotations to literature abstracts or full-text articles (Wei, Kao & Lu, 2013; Kim & Wang, 2012). While these methods, tools, and repositories are not commonly designed to normalize mentions of biological entities to a knowledge graph, we demonstrate here how a normalization of text to a knowledge graph can be achieved, and subsequently use the combined information in our multi-modal machine learning approach. Consequently, our method has the potential to increase the value of freely available Linked Data resources and connect them directly to the methods and tools developed for natural language processing and text mining in biology and biomedicine.

One potential objection to using features generated from the biomedical literature is that the association between a drug and its target or indication may already be stated explicitly in the literature and could therefore be extracted more easily by methods relying on text mining and natural language processing. We tested how many drugs co-occur with their targets or indications in our literature-derived corpus compared to the total number of co-occurrences between mentions of drugs and proteins or diseases. Among all of the directly co-occurring mentions of drugs and proteins and drugs and diseases in the abstracts, 2.8% and 0.8% are positive pairs in our drug–target and drug–indication set, respectively. However, among the positive pairs that are both found in literature and the knowledge graph, the directly co-occurring drug–target pairs are 27.3% and drug–disease pairs 63.4%. We experimented with removing all abstracts in which the drug and protein or drug and disease pairs that are in our evaluation set co-occur. Table S6 shows the resulting performance and demonstrates that removing the directly co-occurring pairs does not change results significantly.

## Conclusion

We developed a generic method for combining information in knowledge graphs and natural language texts, and jointly learns both. This method is capable of utilizing information in a knowledge graph as background knowledge when “reading” text and vice versa when learning from structured information in a knowledge graph. We demonstrate that our method can be used to predict DTI and indications.

In the future, it would be beneficial to develop better entity normalization methods that can directly normalize entity mentions in text to a knowledge graph. We also intend to evaluate the success of our approach on full-text articles so that more information, in particular regarding methods and experimental protocols, can be utilized by our approach. Methodologically, we also intend to apply other knowledge graph embedding methods, in particular translational embeddings (Bordes et al., 2013; Nickel, Rosasco & Poggio, 2016; Dai & Yeung, 2006), that have previously been combined successfully with textual information (Wang et al., 2014c), and evaluate their performance for prediction of biological relations.

## Supplemental Information

10.7717/peerj.13061/supp-1 Supplemental Information 1 Supplementary Tables

## Additional Information and Declarations

### Data Availability

The following information was supplied regarding data availability: The code is available in GitHub: https://github.com/bio-ontology-research-group/multi-drug-embedding. The raw data is available at Zenodo: Alshahrani, Mona. (2022). Combining biomedical knowledge graphs and text to improve predictions for drug-target interactions and drug-indications. [Data set]. Zenodo. https://doi.org/10.5281/zenodo.6148694.

## References

[ref-1] Agarwal P, Searls DB (2008). Literature mining in support of drug discovery. Briefings in Bioinformatics.

[ref-2] Ali M, Hoyt CT, Domingo-Fernandez D., Lehmann J, Jabeen H (2018). BioKEEN: a library for learning and evaluating biological knowledge graph embeddings. bioRxiv.

[ref-3] Alshahrani M, Hoehndorf R (2018). Semantic disease gene embeddings (SmuDGE): phenotype-based disease gene prioritization without phenotypes. Bioinformatics.

[ref-4] Alshahrani M, Khan MA, Maddouri O, Kinjo AR, Queralt-Rosinach N, Hoehndorf R (2017). Neuro-symbolic representation learning on biological knowledge graphs. Bioinformatics.

[ref-5] Alshahrani M, Thafar MA, Essack M. (2021). Application and evaluation of knowledge graph embeddings in biomedical data. PeerJ Computer Science.

[ref-6] Andronis C, Sharma A, Virvilis V, Deftereos S, Persidis A (2011). Literature mining, ontologies and information visualization for drug repurposing. Briefings in Bioinformatics.

[ref-7] Angermueller C, Pärnamaa T, Parts L, Stegle O (2016). Deep learning for computational biology. Molecular Systems Biology.

[ref-8] Ashburner M, Ball CA, Blake JA, Botstein D, Butler H, Cherry MJ, Davis AP, Dolinski K, Dwight SS, Eppig JT, Harris MA, Hill DP, Tarver LI, Kasarskis A, Lewis S, Matese JC, Richardson JE, Ringwald M, Rubin GM, Sherlock G (2000). Gene ontology: tool for the unification of biology. Nature Genetics.

[ref-9] Beckett D (2004). RDF/XML Syntax Specification (Revised). W3C recommendation, World Wide Web Consortium (W3C).

[ref-10] Belleau F, Nolin M-A, Tourigny N, Rigault P, Morissette J (2008). Bio2RDF: towards a mashup to build bioinformatics knowledge systems. Journal of Biomedical Informatics.

[ref-11] Berners-Lee T, Hendler J, Lassila O (2001). The semantic web. Scientific American.

[ref-12] Bertinetto L, Valmadre J, Henriques JF, Vedaldi A, Torr PH (2016). Fully-convolutional siamese networks for object tracking.

[ref-13] Bizer C, Heath T, Berners-Lee T (2011). Linked data: The story so far. Semantic services, interoperability and web applications: emerging concepts.

[ref-14] Bordes A, Usunier N, Garcia-Duran A, Weston J, Yakhnenko O, Burges CJC, Bottou L, Welling M, Ghahramani Z, Weinberger KQ (2013). Translating embeddings for modeling multi-relational data. Advances in neural information processing systems 26.

[ref-15] Brickley D, Guha RV (2004). RDF vocabulary description language 1.0: RDF schema. https://www.w3.org/2001/sw/RDFCore/Schema/200212bwm/.

[ref-16] Callahan A, Cruz-Toledo J, Ansell P, Dumontier M (2013). Bio2RDF release 2: improved coverage, interoperability and provenance of life science linked data.

[ref-17] Chen L, Zeng W-M, Cai Y-D, Feng K-Y, Chou K-C (2012). Predicting anatomical therapeutic chemical (ATC) classification of drugs by integrating chemical-chemical interactions and similarities. PLOS ONE.

[ref-18] Chen X, Yan CC, Zhang X, Zhang X, Dai F, Yin J, Zhang Y (2015). Drug–target interaction prediction: databases, web servers and computational models. Briefings in Bioinformatics.

[ref-19] Dai G, Yeung D-Y (2006). Tensor embedding methods.

[ref-20] Devlin J, Chang M-W, Lee K, Toutanova K (2018). Bert: pre-training of deep bidirectional transformers for language understanding.

[ref-21] Dietz L, Kotov A, Meij E (2018). Utilizing knowledge graphs for text-centric information retrieval.

[ref-22] Ehrlinger L, Wöß W (2016). Towards a definition of knowledge graphs.

[ref-23] Ezzat A, Wu M, Li X-L, Kwoh C-K (2018). Computational prediction of drug-target interactions using chemogenomic approaches: an empirical survey. Briefings in Bioinformatics.

[ref-24] Fawcett T (2006). An introduction to ROC analysis. Pattern Recognition Letters.

[ref-25] Frijters R, Van Vugt M, Smeets R, Van Schaik R, De Vlieg J, Alkema W (2010). Literature mining for the discovery of hidden connections between drugs, genes and diseases. PLOS Computational Biology.

[ref-26] Fu G, Ding Y, Seal A, Chen B, Sun Y, Bolton E (2016). Predicting drug target interactions using meta-path-based semantic network analysis. BMC Bioinformatics.

[ref-27] Gulli A, Pal S (2017). Deep learning with Keras.

[ref-28] Gutiérrez-Basulto V, Schockaert S (2018). From knowledge graph embedding to ontology embedding: region based representations of relational structures.

[ref-29] Gysi DM, Do Valle Í, Zitnik M, Ameli A, Gan X, Varol O, Ghiassian SD, Patten J, Davey Robert ALJ, Barabasi A-L (2021). Network medicine framework for identifying drug-repurposing opportunities for COVID-19. Proceedings of the National Academy of Sciences of the United States of America.

[ref-30] Hinton G, Srivastava N, Swersky K (2012). Lecture 6a overview of mini–batch gradient descent. https://www.youtube.com/watch?v=tCTfb6PAr4w.

[ref-31] Hoehndorf R, Schofield PN, Gkoutos GV (2015). Analysis of the human diseasome using phenotype similarity between common, genetic, and infectious diseases. Scientific Reports.

[ref-32] Hoffmann R, Zhang C, Ling X, Zettlemoyer L, Weld DS (2011). Knowledge-based weak supervision for information extraction of overlapping relations.

[ref-33] Ji S, Pan S, Cambria E, Marttinen P, Philip SY (2021). A survey on knowledge graphs: representation, acquisition, and applications. IEEE Transactions on Neural Networks and Learning Systems.

[ref-34] Jupp S, Malone J, Bolleman J, Brandizi M, Davies M, Garcia L, Gaulton A, Gehant S, Laibe C, Redaschi N, Wimalaratne SM, Martin M, Le Novre N, Parkinson H, Birney E, Jenkinson AM (2014a). The EBI RDF platform: linked open data for the life sciences. Bioinformatics.

[ref-35] Jupp S, Malone J, Bolleman J, Brandizi M, Davies M, Garcia L, Gaulton A, Gehant S, Laibe C, Redaschi N, Wimalaratne SM, Martin M, Novére NL, Parkinson H, Birney E, Jenkinson AM (2014b). The EBI RDF platform: linked open data for the life sciences. Bioinformatics.

[ref-36] Jupp S, Stevens R, Hoehndorf R (2012). Logical Gene Ontology Annotations (GOAL): exploring gene ontology annotations with OWL. Journal of Biomedical Semantics.

[ref-37] Kim J-D, Wang Y (2012). PubAnnotation: a persistent and sharable corpus and annotation repository.

[ref-38] Köhler S, Doelken SC, Mungall CJ, Bauer S, Firth HV, Bailleul-Forestier I, Black GCM, Brown DL, Brudno M, Campbell J, FitzPatrick DR, Eppig JT, Jackson AP, Freson K, Girdea M, Helbig I, Hurst JA, Jähn J, Jackson LG, Kelly AM, Ledbetter DH, Mansour S, Martin CL, Moss C, Mumford A, Ouwehand WH, Park S-M, Riggs ER, Scott RH, Sisodiya S, Vooren SV, Wapner RJ, Wilkie AOM, Wright CF, Vulto-van Silfhout AT, Leeuw Nd, de Vries BBA, Washingthon NL, Smith CL, Westerfield M, Schofield P, Ruef BJ, Gkoutos GV, Haendel M, Smedley D, Lewis SE, Robinson PN (2014). The Human Phenotype Ontology project: linking molecular biology and disease through phenotype data. Nucleic Acids Research.

[ref-39] Kuhn M, Letunic I, Jensen LJ, Bork P (2015). The SIDER database of drugs and side effects. Nucleic Acids Research.

[ref-40] Kuhn M, Szklarczyk D, Franceschini A, von Mering C, Jensen LJ, Bork P (2012). STITCH 3: zooming in on protein-chemical interactions. Nucleic Acids Research.

[ref-41] Leaman R, Gonzalez G (2008). BANNER: an executable survey of advances in biomedical named entity recognition.

[ref-42] Leaman R, Islamaj Doğan R, Lu Z (2013). DNorm: disease name normalization with pairwise learning to rank. Bioinformatics.

[ref-43] Leaman R, Wei C-H, Lu Z (2015). tmChem: a high performance approach for chemical named entity recognition and normalization. Journal of Cheminformatics.

[ref-44] LeCun Y, Bengio Y, Hinton G (2015). Deep learning. Nature.

[ref-45] Lee J, Yoon W, Kim S, Kim D, Kim S, So CH, Kang J (2020). BioBERT: a pre-trained biomedical language representation model for biomedical text mining. Bioinformatics.

[ref-46] Li Y, Li J, Bian N (2019). DNILMF-LDA: prediction of lncRNA-disease associations by dual-network integrated logistic matrix factorization and Bayesian optimization. Genes.

[ref-47] Lin Y, Liu Z, Sun M, Liu Y, Zhu X (2015). Learning entity and relation embeddings for knowledge graph completion. AAAI.

[ref-48] Luo Y, Zhao X, Zhou J, Yang J, Zhang Y, Kuang W, Peng J, Chen L, Zeng J (2017). A network integration approach for drug-target interaction prediction and computational drug repositioning from heterogeneous information. Nature Communications.

[ref-49] Mei J-P, Kwoh C-K, Yang P, Li X-L, Zheng J (2013). Drug–target interaction prediction by learning from local information and neighbors. Bioinformatics.

[ref-50] Mikolov T, Sutskever I, Chen K, Corrado GS, Dean J (2013). Distributed representations of words and phrases and their compositionality.

[ref-51] Mohamed SK, Nováček V, Nounu A (2019). Discovering protein drug targets using knowledge graph embeddings. Bioinformatics.

[ref-52] Mohamed SK, Nováček V, Nounu A (2020). Discovering protein drug targets using knowledge graph embeddings. Bioinformatics.

[ref-53] Muñoz E, Nováček V, Vandenbussche P-Y (2017). Facilitating prediction of adverse drug reactions by using knowledge graphs and multi-label learning models. Briefings in Bioinformatics.

[ref-54] Nair V, Hinton GE (2010). Rectified linear units improve restricted boltzmann machines.

[ref-55] Nascimento AC, Prudêncio RB, Costa IG (2016). A multiple kernel learning algorithm for drug-target interaction prediction. BMC Bioinformatics.

[ref-56] Nelson W, Zitnik M, Wang B, Leskovec J, Goldenberg A, Sharan R (2019). To embed or not: network embedding as a paradigm in computational biology. Frontiers in Genetics.

[ref-57] Nickel M, Murphy K, Tresp V, Gabrilovich E (2016). A review of relational machine learning for knowledge graphs. Proceedings of the IEEE.

[ref-58] Nickel M, Rosasco L, Poggio T (2016). Holographic embeddings of knowledge graphs.

[ref-59] Paulheim H (2017). Knowledge graph refinement: a survey of approaches and evaluation methods. Semantic Web.

[ref-60] Pedregosa F, Varoquaux G, Gramfort A, Michel V, Thirion B, Grisel O, Blondel M, Prettenhofer P, Weiss R, Dubourg V, Vanderplas J, Passos A, Cournapeau D, Brucher M, Perrot M, Duchesnay E (2011). Scikit-learn: machine learning in python. Journal of Machine Learning Research.

[ref-61] Pennington J, Socher R, Manning C (2014). Glove: global vectors for word representation.

[ref-62] Percha B, Altman RB (2018). A global network of biomedical relationships derived from text. Bioinformatics.

[ref-63] Perozzi B, Al-Rfou R, Skiena S (2014). Deepwalk: online learning of social representations.

[ref-64] Piñero J, Bravo À, Queralt-Rosinach N, Gutiérrez-Sacristán A, Deu-Pons J, Centeno E, García-García J, Sanz F, Furlong LI (2016). DisGeNET: a comprehensive platform integrating information on human disease-associated genes and variants. Nucleic Acids Research.

[ref-65] Pryor R, Cabreiro F (2015). Repurposing metformin: an old drug with new tricks in its binding pockets. Biochemical Journal.

[ref-66] Raví D, Wong C, Deligianni F, Berthelot M, Andreu-Perez J, Lo B, Yang GZ (2017). Deep learning for health informatics. IEEE Journal of Biomedical and Health Informatics.

[ref-67] Rebholz-Schuhmann D, Oellrich A, Hoehndorf R (2012). Text-mining solutions for biomedical research: enabling integrative biology. Nature Reviews Genetics.

[ref-68] Ristoski P, Paulheim H, Groth P, Simperl E, Gray A, Sabou M, Krötzsch M, Lecue F, Flöck F, Gil Y (2016). RDF2Vec: RDF graph embeddings for data mining. The Semantic Web –ISWC 2016.

[ref-69] Sang S, Yang Z, Wang L, Liu X, Lin H, Wang J (2018). SemaTyP: a knowledge graph based literature mining method for drug discovery. BMC Bioinformatics.

[ref-70] Schriml LM, Arze C, Nadendla S, Chang Y-WW, Mazaitis M, Felix V, Feng G, Kibbe WA (2011). Disease ontology: a backbone for disease semantic integration. Nucleic Acids Research.

[ref-71] Seal A, Ahn Y-Y, Wild DJ (2015). Optimizing drug–target interaction prediction based on random walk on heterogeneous networks. Journal of Cheminformatics.

[ref-72] Smaili FZ, Gao X, Hoehndorf R (2018). Onto2Vec: joint vector-based representation of biological entities and their ontology-based annotations. Bioinformatics.

[ref-73] Smaili FZ, Gao X, Hoehndorf R (2019). Opa2vec: combining formal and informal content of biomedical ontologies to improve similarity-based prediction. Bioinformatics.

[ref-74] Swanson DR (1990). Medical literature as a potential source of new knowledge. Bulletin of the Medical Library Association.

[ref-75] Szklarczyk D, Franceschini A, Kuhn M, Simonovic M, Roth A, Minguez P, Doerks T, Stark M, Muller J, Bork P, Jensen LJ, Mering Cv (2010). The STRING database in 2011: functional interaction networks of proteins, globally integrated and scored. Nucleic Acids Research.

[ref-76] Thafar M, Raies AB, Albaradei S, Essack M, Bajic VB (2019). Comparison study of computational prediction tools for drug-target binding affinities. Frontiers in Chemistry.

[ref-77] Thafar MA, Albaradie S, Olayan RS, Ashoor H, Essack M, Bajic VB (2020a). Computational drug-target interaction prediction based on graph embedding and graph mining.

[ref-78] Thafar MA, Olayan RS, Albaradei S, Bajic VB, Gojobori T, Essack M, Gao X (2021). DTi2Vec: Drug–target interaction prediction using network embedding and ensemble learning. Journal of Cheminformatics.

[ref-79] Thafar MA, Olayan RS, Ashoor H, Albaradei S, Bajic VB, Gao X, Gojobori T, Essack M (2020b). DTiGEMS+: drug–target interaction prediction using graph embedding, graph mining, and similarity-based techniques. Journal of Cheminformatics.

[ref-80] The UniProt Consortium (2016). UniProt: the universal protein knowledgebase. Nucleic Acids Research.

[ref-81] UniProt Consortium T (2018). UniProt: the universal protein knowledgebase. Nucleic Acids Research.

[ref-82] Van der Maaten L, Hinton G (2008). Visualizing Data using t-SNE. Journal of Machine Learning Research.

[ref-83] Wang B, Mezlini AM, Demir F, Fiume M, Tu Z, Brudno M, Haibe-Kains B, Goldenberg A (2014a). Similarity network fusion for aggregating data types on a genomic scale. Nature Methods.

[ref-84] Wang Z, Zhang J, Feng J, Chen Z (2014b). Knowledge graph and text jointly embedding.

[ref-85] Wang Z, Zhang J, Feng J, Chen Z (2014c). Knowledge graph and text jointly embedding.

[ref-86] Wei C-H, Kao H-Y, Lu Z (2013). PubTator: a web-based text mining tool for assisting biocuration. Nucleic Acids Research.

[ref-87] Wei C-H, Kao H-Y, Lu Z (2015). GNormPlus: an integrative approach for tagging genes, gene families, and protein domains. BioMed Research International.

[ref-88] Williams AJ, Harland L, Groth P, Pettifer S, Chichester C, Willighagen EL, Evelo CT, Blomberg N, Ecker G, Goble C, Mons B (2012). Open PHACTS: semantic interoperability for drug discovery. Drug Discovery Today.

[ref-89] Wishart DS, Knox C, Guo AC, Cheng D, Shrivastava S, Tzur D, Gautam B, Hassanali M (2008). DrugBank: a knowledgebase for drugs, drug actions and drug targets. Nucleic Acids Research.

[ref-90] Xie R, Liu Z, Jia J, Luan H, Sun M (2016). Representation learning of knowledge graphs with entity descriptions.

[ref-91] Yamanishi Y, Araki M, Gutteridge A, Honda W, Kanehisa M (2008). Prediction of drug–target interaction networks from the integration of chemical and genomic spaces. Bioinformatics.

