# Supplementary material for: Combining biomedical knowledge graphs and text to improve predictions for drug-target interactions and drug-indications
Source: PeerJ. 2022 Apr 4;10:e13061. doi: 10.7717/peerj.13061 (PMC8988936; doi:10.7717/peerj.13061)
Supplement: Supplemental Information 1 [file peerj-10-13061-s001.pdf]

Supplementary materials: Combining Biomedical Knowledge Graphs and Text to Improve Predictions for Drug-Target Interactions and Drug-Indications

Mona Alshahrani, Abdullah Almansour, Asma Alkhaldi, Maha Thafar, Mahmut Uludag, Magbubah Essack, and Robert Hoehndorf

Number of walks<br>Walks lengths	50	100	150
5	0.889	0.891	0.895
10	0.882	0.885	0.883
15	0.879	0.879	0.878
20	0.883	0.881	0.881

Table 1: AUROC results for prediction of drug indications based on the knowledge graph corpus while varying then number of walks and walks lengths.

Hidden Units<br>One layer	32	64	128	256	512
Knowledge graph	0.874	0.876	0.878	0.880	0.882
Pubmed abstracts	0.867	0.876	0.877	0.880	0.883
Concatenated embeddings	0.887	0.893	0.898	0.901	0.899
Concatenated corpus	0.879	0.879	0.890	0.896	0.897
Hidden Units<br>Two layers	32	64	128	256	512
Knowledge graph	0.868	0.875	0.878	0.882	0.882
Pubmed abstracts	0.866	0.874	0.878	0.877	0.877
Concatenated embeddings	0.888	0.895	0.899	0.901	0.895
Concatenated corpus	0.879	0.882	0.888	0.889	0.879

Table 2: AUROC results for prediction of drug targets with different embeddings methods and neural network architectures.

Hidden Units<br>One layer	32	64	128	256	512
Knowledge graph	0.869	0.875	0.881	0.883	0.888
Pubmed abstracts	0.909	0.919	0.928	0.927	0.929
Concatenated embeddings	0.903	0.909	0.913	0.916	0.914
Concatenated corpus	0.914	0.921	0.924	0.929	0.932
Hidden Units<br>Two layers	32	64	128	256	512
Knowledge graph	0.862	0.862	0.874	0.876	0.885
Pubmed abstracts	0.899	0.904	0.921	0.924	0.920
Concatenated embeddings	0.898	0.905	0.912	0.910	0.918
Concatenated corpus	0.897	0.909	0.920	0.920	0.918

Table 3: AUROC results for prediction of drug indications with different embeddings methods and neural network architectures.

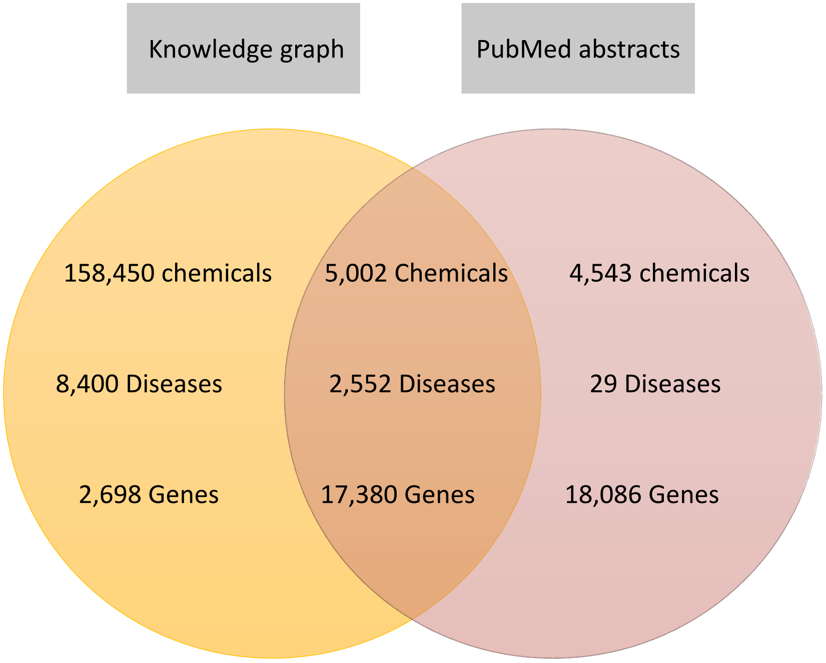

Figure 1: Overlap between entities recognized in text and contained in our knowledge graph.

Source	# of drugs	# of genes	# of DTIs
Knowledge graph	1,466	20,068	
MEDLINE (PubTator) corpus	9,545	35,466	
Knowledge Graph and PubMed	932	17,380	
Evaluation set (STITCH)	98,567	9,782	432,512
Knowledge Graph, PubMed and evaluation set	820	7,201	40,862

Table 4: The number of drugs, genes, and their associations in our knowledge graph (after removing *has-target edges*), MEDLINE abstracts in the PubTator corpus, in the evaluation set, and the overlap between all resources.

Source	# of drugs	# of diseases	# of associations
Knowledge graph	163,420	10,952	
MEDLINE (PubTator) corpus	9,545	2,581	
Knowledge Graph and PubMed	4,993	2,552	
Evaluation set (SIDER indications)	1,224	871	6,704
Knowledge Graph, PubMed and evaluation set	754	664	3,977

Table 5: The number of drugs, diseases, and their associations in our knowledge graph (after removing *has-indication edges*), MEDLINE abstracts in the PubTator corpus, in the evaluation set, and the overlap between all resources.

	drug-targets	drug-indications
PubMed Abstracts	0.868	0.904
Concatenated embeddings	0.898	0.927
Concatenated corpus	0.866	0.903

Table 6: AUROC results for prediction of drug targets and drug indications after explicitly removing all the abstract that contain co-occurrences.

Table 7: Performance results for predicting drug–target associations, based on our five embeddings approaches and using three classification models (Artificial Neural Networks (ANN), Random Forest (RF) and Logistic regression (LR)).

Model	Embedding Method	ROCAUC	Average recall @100	Average Rec[illegible]
**ANN**	Knowledge graph (Walking RDF/OWL)	0.882	0.37	0.09
	Knowledge graph (TransE)	0.873	0.31	0.04
	PubMed abstracts	0.883	0.47	0.14
	Concatenated embeddings	0.901	0.49	0.14
	Concatenated corpus	0.897	0.50	0.14
**RF**	Knowledge graph (Walking RDF/OWL)	0.860	0.36	0.08
	Knowledge graph (TransE)	0.859	0.31	0.05
	PubMed abstracts	0.852	0.49	0.18
	Concatenated embeddings	0.867	0.45	0.13
	Concatenated corpus	0.879	0.51	0.18
**LR**	Knowledge graph (Walking RDF/OWL)	0.840	0.11	0.03
	Knowledge graph (TransE)	0.829	0.11	0.02
	PubMed abstracts	0.832	0.21	0.05
	Concatenated embeddings	0.858	0.23	0.07
	Concatenated corpus	0.841	0.22	0.06

Table 8: Prediction performance for drug-disease associations linked by indications, based on five approaches, using three classification models (Artificial Neural Networks (ANN), Random Forest (RF) and Logistic regression (LR)).

Model	Embedding Method	ROCAUC	Average recall@100	Average recal[illegible]
**ANN**	Knowledge graph (Walking RDF/OWL)	0.888	0.47	0.12
	Knowledge graph (TransE)	0.866	0.38	0.05
	PubMed abstracts	0.928	0.63	0.26
	Concatenated embeddings	0.916	0.61	0.23
	Concatenated corpus	0.932	0.64	0.25
**RF**	Knowledge graph (Walking RDF/OWL)	0.895	0.44	0.13
	Knowledge graph (TransE)	0.888	0.43	0.11
	PubMed abstracts	0.912	0.61	0.24
	Concatenated embeddings	0.908	0.54	0.17
	Concatenated corpus	0.918	0.60	0.22
**LR**	Knowledge graph (Walking RDF/OWL)	0.842	0.30	0.06
	Knowledge graph (TransE)	0.836	0.31	0.03
	PubMed abstracts	0.846	0.40	0.11
	Concatenated embeddings	0.862	0.39	0.09
	Concatenated corpus	0.858	0.39	0.12

**Drug**	**Target (Entrez ID)**	**Knowledge graph**	**PubMed abstracts**	**Concatenated embeddings**	**Concatenated corpus**
Megestrol acetate (CID00004048)	2908	ranked 13	ranked 10	ranked 6	**ranked 4**
Propantheline (CID00004934)	1131	ranked 91	ranked 13	**ranked 1**	**ranked 1**
Dothiepin (CID00003155)	1129	ranked 62	ranked 26	ranked 19	**ranked 1**
Paclitaxel (CID00004666)	7157	ranked 5	ranked 3	ranked 5	**ranked 2**
Cortisol (CID00003640)	1551	ranked 13	ranked 20	**ranked 3**	ranked 10
Omeprazole (CID00004594)	1544	ranked 53	ranked 18	ranked 7	**ranked 2**

Table 9: The predicted ranks of different embeddings approaches in drug-target predictions

**Drug**	**Indication**	**Knowledge graph**	**PubMed abstracts**	**Concatenated embeddings**	**Concatenated corpus**
Cetirizine (CID00002678)	allergic hypersensitivity disease (DOID:1205)	ranked 34	ranked 4	**ranked 1**	ranked 10
Etoposide (CID00003310)	leukemia (DOID:1240)	ranked 177	ranked 3	ranked 11	**ranked 1**
Ramiprilat (CID05464096)	cerebrovascular disease (DOID:6713)	ranked 76	**ranked 1**	**ranked 1**	ranked 3
Clindamycin (CID00002786)	impetigo (DOID:8504)	ranked 16	ranked 11	**ranked 1**	**ranked 1**
Cefuroxime (CID00002658)	pneumonia (DOID:552)	ranked 46	ranked 7	ranked 3	**ranked 1**
Metformin (CID00004091)	diabetes mellitus (DOID:9351)	ranked 3	ranked 6	**ranked 1**	ranked 3

Table 10: The predicted ranks of different embeddings approaches in drug-indication predictions
